# Building an organic computing device with multiple interconnected brains

**DOI:** 10.1038/srep11869

**Published:** 2015-07-09

**Authors:** Miguel Pais-Vieira, Gabriela Chiuffa, Mikhail Lebedev, Amol Yadav, Miguel A. L. Nicolelis

**Affiliations:** 1 Department of Neurobiology, Duke University, Durham, North Carolina 27710; 2 Department of Biomedical Engineering, Duke University, Durham, North Carolina 27710; 3 Department of Psychology and Neuroscience, Duke University, Durham, North Carolina 27710; 4 Duke Center for Neuroengineering, Duke University, Durham, North Carolina 27710; 5 Edmond and Lily Safra International Institute for Neuroscience of Natal, Natal, Brazil

## Abstract

Recently, we proposed that Brainets, i.e. networks formed by multiple animal brains, cooperating and exchanging information in real time through direct brain-to-brain interfaces, could provide the core of a new type of computing device: an organic computer. Here, we describe the first experimental demonstration of such a Brainet, built by interconnecting four adult rat brains. Brainets worked by concurrently recording the extracellular electrical activity generated by populations of cortical neurons distributed across multiple rats chronically implanted with multi-electrode arrays. Cortical neuronal activity was recorded and analyzed in real time, and then delivered to the somatosensory cortices of other animals that participated in the Brainet using intracortical microstimulation (ICMS). Using this approach, different Brainet architectures solved a number of useful computational problems, such as discrete classification, image processing, storage and retrieval of tactile information, and even weather forecasting. Brainets consistently performed at the same or higher levels than single rats in these tasks. Based on these findings, we propose that Brainets could be used to investigate animal social behaviors as well as a test bed for exploring the properties and potential applications of organic computers.

After introducing the concept of brain-to-brain interfaces (BtBIs)^1^, our laboratory demonstrated experimentally that BtBIs could be utilized to directly transfer tactile or visuomotor information between pairs of rat brains in real time^2^. Since our original report, other studies have highlighted several properties of BtBIs^1^,^3^, such as transmission of hippocampus representations between rodents^4^, transmission of visual information between a human and a rodent^5^, and transmission of motor information between two humans^6^,^7^. Our lab has also shown that Brainets could allow monkey pairs or triads to perform cooperative motor tasks mentally by inducing, accurate synchronization of neural ensemble activity across individual brains^8^.

In addition to the concept of BtBIs, we have also suggested that networks of multiple interconnected animal brains, which we dubbed Brainet^1^, could provide the core for a new type of computing device: an organic computer. Here, we tested the hypothesis that such a Brainet could potentially exceed the performance of individual brains, due to a distributed and parallel computing architecture^1^,^8^. This hypothesis was tested by constructing a Brainet formed by four interconnected rat brains and then investigating how it could solve fundamental computational problems (Fig. 1A–C). In our Brainet, all four rats were chronically implanted with multielectrode arrays, placed bilaterally in the primary somatosensory cortex (S1). These implants were used to both record neural ensemble electrical activity and transmit virtual tactile information via intracortical electrical microstimulation (ICMS). Once animals recovered from the implantation surgery, the resulting 4-rat Brainets (Fig. 1) were tested in a variety of ways. Our central goal was to investigate how well different Brainet architectures could be employed by the four rats to collaborate in order to solve a particular computational task. Different Brainet designs were implemented to address three fundamental computational problems: discrete classification, sequential and parallel computations, and memory storage/retrieval^1^. As predicted, we observed that Brainets consistently outperformed individual rats in each of these tasks.

## Results

All experiments with 4-rat Brainets were pooled from a sample of 16 animals that received cortical implants from which we could simultaneously record the extracellular activity from 15–66 S1 neurons per Brainet (total of 2,738 neurons recorded across 71 sessions).

### Brainet for neural synchronization

**Figure 1:**
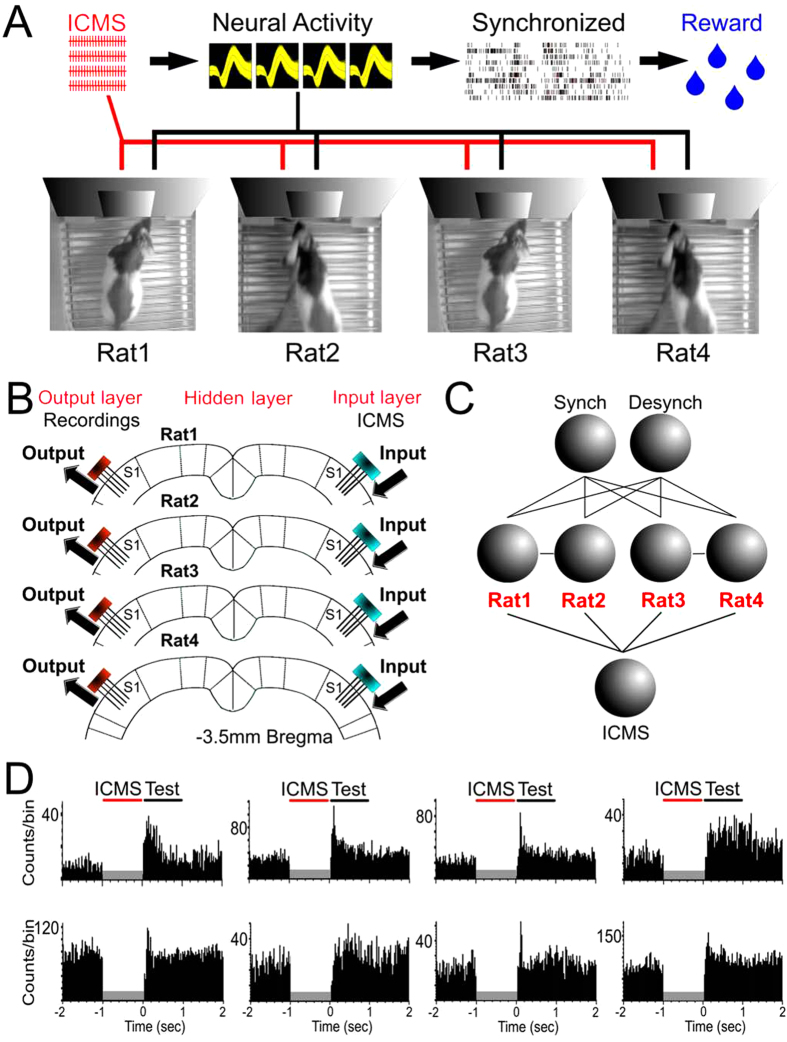
Experimental apparatus scheme for a Brainet computing device. **A**) A Brainet of four interconnected brains is shown. The arrows represent the flow of information through the Brainet. Inputs were delivered as simultaneous ICMS patterns to the S1 cortex of each rat. Neural activity was then recorded and analyzed in real time. Rats were required to synchronize their neural activity with the remaining of the Brainet to receive water **B**) Inputs to the Brainet were delivered as ICMS patterns to the left S1, while outputs were calculated using the neural responses recorded from the right S1. **C**) Brainet architectures were set to mimic hidden layers of an artificial neural network. **D**) Examples of perievent histograms of neurons after the delivery of ICMS.

**Figure 2:**
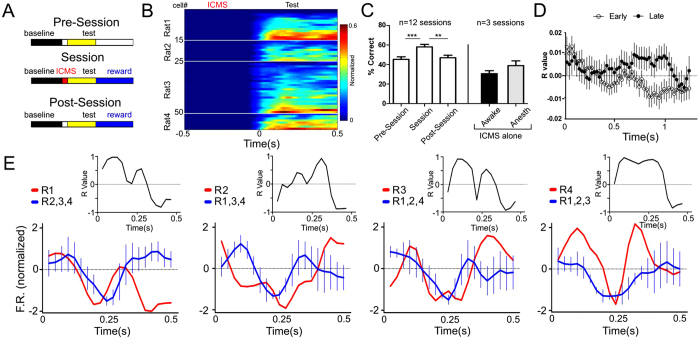
The Brainet can synchronize neural activity. **A**) The different colors indicate the different manipulations used to study synchronization across the network. During the pre-session, rats were tested for periods of spurious neural synchronization. No ICMS or rewards were delivered here. During sessions, rats were tested for increased neural synchronization due to detection of the ICMS stimulus (red period). Successful synchronization was rewarded with water. During the post session, rats were tested for periods of neural synchronization due to the effects of reward (e.g. continuous whisking/licking). Successful synchronization was rewarded with water, but no ICMS stimulus was delivered. **B**) Example of neuronal activity across the Brainet. After the ICMS there was a general tendency for neural activity to increase. Periods of maximum firing rate are represented in red. **C**) The performance of the Brainet during sessions was above the pre-sessions and post-sessions. Also, delivery of ICMS alone or during anesthetized states also resulted in poor performances. ** and *** indicate P < 0.01 and P < 0.0001 respectively. **D**) Overall changes in R values in early and late sessions show that improvements in performances were accompanied by specific changes in the periods of synchronized activity. **E**) Example of a synchronization trial. The lower panels show, in red, the neural activity of each rat and, in blue, the average of neural activity for the remaining of the Brainet. The upper panels depict the R value for the correlation coefficient between each rat and the remaining of the Brainet. There was an overall tendency for the Brainet to correlate in the beginning of the test period.

Rats were water deprived and trained on a task that required them to synchronize their neural activity after an ICMS stimulus. A total of six rats were used in 12 sessions to run this first experiment. As depicted in Fig. 1A–C, the processing chain in these experiments started with the simultaneous delivery of an ICMS pattern to one of the S1 cortices of all subjects, then processing of tactile information with a single-layer Brainet, followed by generation of the system output by the contralateral S1 cortex of each animal. Each trial was comprised of four epochs: waiting (baseline), ICMS delivery, test, and reward. ICMS patterns (20 pulses at 22–26 Hz) were unilaterally delivered to the S1 of each rat. Neuronal responses to the ICMS were evaluated during the test period when S1 neuronal ensemble activity was sampled from the hemisphere contralateral to the stimulation site (Figs. 1D and 2A–E) (Fig. 2A–E). Rats were rewarded if their cortical activity became synchronized during the test period. The correlation coefficient R was used as the measure of global Brainet synchrony. Thus, R measured the linear correlation between the normalized firing rate of all neurons in a given rat and the average normalized firing rate for all neurons recorded in the remaining three rats (see Methods for details). If at least three rats presented R values greater or equal to 0.2, a trial was considered successful, and all four rats were rewarded. Otherwise no reward was given to any rat. Two conditions served as controls: the pre-session, where no ICMS or water reward were delivered, and the post-session, where no ICMS was delivered but rats were still rewarded if they satisfied the correlation criterion (Fig. 2A).

Behaviorally, rats remained mostly calm or immobile during the baseline period. After the ICMS pattern was delivered simultaneously to all animals, rats typically displayed periods of whisking and licking movements. A sample of S1 neuronal population activity during this period is shown in Fig. 2B (also see Fig. 1D for examples of individual neurons perievent histograms). Typically, after the delivery of ICMS, there was a sharp decrease in the neuronal firing rate of the neurons (~20 ms), followed by a sudden firing rate increase (~100 ms). While the main measure of accuracy for this task was the degree in which cortical neuronal populations fired synchronously, it is important to emphasize that the build up of these ensemble firing patterns depended highly on how single S1 neurons modulated their firing rate as a result of electrical microstimulation. Thus, ICMS served as a reset signal that allowed rats to synchronize their neural activity to the remaining network (Fig. 2D,E). Note that, in this task, rats were not exchanging neural information through the BtBI. Instead the timing of the ICMS stimulus, the partial contact allowed through the Plexiglas panels, and the reward were the only sources of information available for rats to succeed in the task.

As the Brainet consistently exhibited the best performance during the first trials, we focused our subsequent analysis on the first 30-trial block of each session. Overall, this 4-rat Brainet was able to synchronize the neural activity of the constituent rats significantly above Pre-Session (Brainet: 57.95 ± 2%; Pre-Sessions: 45.95 ± 2%; F_2,24_ = 10.99; P = 0.0004; Dunnett’s test: P < 0.001) and Post-Session levels (46.41 ± 2%; Dunnett’s test: P < 0.01; Fig. 2C).

Over approximately 1.5 weeks (total of 12 sessions), this Brainet gradually improved its performance, from 54.76 ± 3.16% (mean ± standard error; the first 6 days) to 61.67 ± 3.01% correct trials (the last 6 days; F_1,2_ = 5.770, P = 0.0175 for interaction; Bonferroni post hoc comparisons: pre vs session initial start P > 0.05; pre vs session end P < 0.01; session vs post start P > 0.05; session vs post end P < 0.001). The high fidelity of information transfer in this Brainet configuration was further confirmed by the observation that the performance of individual rats reached 65.28 ± 1.70%. In other words, a 4-rat Brainet was capable of maintaining a level of global neuronal synchrony across multiple brains that was virtually identical to that observed in the cortex of a single rat (Brainet level = 61.67 ± 3.07%; Man-Whitney U = 58.0; *P* = 0.4818, n.s.).

A comparison of correlation values between sessions from the first (n = 6) and the last days (n = 6) further demonstrated that daily training on this first task resulted in a statistically significant increase in correlated cortical activity across rats, centered between 700 ms and 1000 ms of the testing period (F = 1.622; df = 1.49; P = 0.0043, Fig. 2D). The lower panel of Fig. 2E shows the normalized firing rate for each rat (in red) and for the remaining Brainet (in blue) in one trial. The upper panels show R value changes for the correlation between neuronal activity in each rat and the remaining Brainet. Notice the overall tendency for most rats to increase the R values soon after the delivery of the ICMS pattern (T = 0 seconds).

To determine if reward was mandatory for the correlation to emerge in the Brainet, we performed three control sessions with awake animals receiving ICMS (but no reward). The performances dropped to levels below chance (performance: 30.67 ± 3.0%; see Fig. 2C). Further, in another three sessions where ICMS was applied to anesthetized animals, the Brainet performed close to chance levels again (performance: 38.89 ± 4.8%; see Fig. 2C). These results demonstrated that the Brainet could only operate above chance in awake behaving rats in which there was an expectation for reward.

After determining that the Brainet could learn to respond to an ICMS input by synchronizing its output across multiple brains, we tested whether such a collective neuronal response could be utilized for multiple computational purposes. These included discrete stimulus classification, storage of a tactile memory, and, by combining the two former tasks, processing of multiple tactile stimuli.

### Brainet for stimulus classification

**Figure 3:**
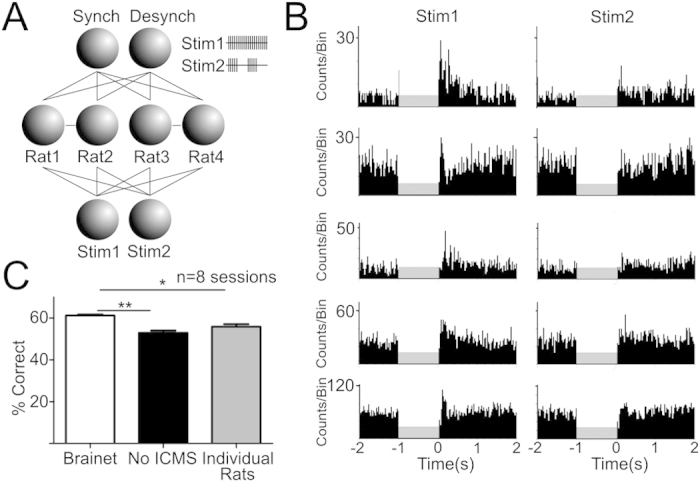
The Brainet can both synchronize and desynchronize neural activity. **A**) Architecture of a Brainet that can synchronize and desynchronize its neural activity to perform virtual tactile stimuli classification. Different patterns of ICMS were simultaneously delivered to each rat in the Brainet. Neural signals from all neurons from each brain were analyzed and compared to the remaining rats in the Brainet. The Brainet was required to synchronize its neural activity to indicate the delivery of a Stimulus 1 and to desynchronize its neural activity to indicate the delivery of a Stimulus 2. **B**) Example of perievent histograms of neurons for ICMS Stimulus 1 and 2. **C**) The Brainet performance was above No-ICMS sessions, and above individual rats’ performances. * indicates P < 0.05; ** indicates P < 0.01; n.s. indicates non significant.

Initially, we trained our 4-rat Brainet to discriminate between two ICMS patterns (Fig. 3A,B, 8 sessions in 4 rats). The first pattern (Stimulus 1) was the same as in the previous experiment (20 pulses at 22–26 Hz), while the second (Stimulus 2) consisted of two separate bursts of four pulses (22–26 Hz). The Brainet was required to report either the presence of Stimulus 1 with an increase in neuronal synchrony across the four rat brains (i.e. R ≥ 0.2 in at least three rats), or Stimulus 2 by a decrease in synchrony (i.e., R < 0.2 in at least three rats). By requiring that the delivery of Stimulus 2 be indicated through a reduction in neuronal synchronization, we further ensured that the Brainet performance was not based on a simple neural response to the ICMS pattern. As in the previous experiment, Stimulus 1 served as a reset signal that allowed rats to synchronize their neural activity to the remaining network. Meanwhile, because Stimulus 2 was much shorter than Stimulus 1, it still induced neural responses in several S1 neurons (Fig. 3B), but its effects were less pronounced and not as likely to induce an overall neural synchronization across the Brainet (see Supplementary Figure 1).

Following training, the Brainet reached an average performance of 61.24 ± 0.5% correct discrimination between Stimuli 1 and 2, which was significantly above No-ICMS sessions (52.97 ± 1.1%, n = 8 sessions; Brainet vs No-ICMS: Dunn’s test: P < 0.01). Moreover, using this more complex task design, the Brainet outperformed individual rats (55.86 ± 1.2%) (Kruskal-Wallis statistic = 10.87, P = 0.0044; Brainet vs Individual Rats; Dunn’s test: P < 0.05; also see Fig. 3C).

**Figure 4:**
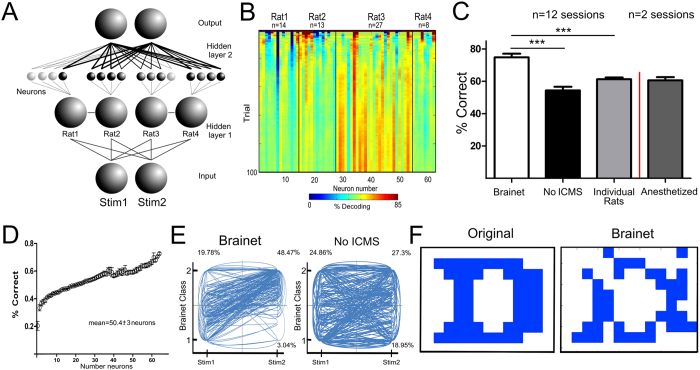
Brainet for discrete classification. **A**) Architecture of a Brainet for stimulus classification. Two different patterns of ICMS were simultaneously delivered to each rat in the Brainet. Neural signals from each individual neuron were analyzed separately and used to determine an overall classification vote for the Brainet. **B**) Example of a session where a total of 62 neurons were recorded from four different animals. Deep blue indicates poor encoding, while dark red indicates good encoding. Although Rat 3 presented the best encoding neurons, all rats contributed to the network’s final classification. **C**) Performance of Brainet during sessions was significantly higher when compared to the No-ICMS sessions. Additionally, because the neural activity is redundant across multiple brains, the overall performance of the Brainet was also higher than in individual brains. *** indicates P < 0.0001. **D**) Neuron dropping curve of Brainet for discrete classification. The effect of redundancy in encoding can be observed in the Brainet as the best encoding cells from each session are removed. **E**) The panels depict the dynamics of the stimulus presented (X axis: 1 or 2) and the Brainet classifications (Y axis: 1 to 2) during sessions and No-ICMS sessions. During regular sessions, the Brainet classifications mostly matched the stimulus presented (lower left and upper right quadrants). Meanwhile, during No ICMS sessions the Brainet classifications were evenly distributed across all four quadrants. The percentages indicate the fraction of trials in each quadrant (Stimulus 1, vote 1 not shown). **F**) Example of an image processed by the Brainet for discrete classification. An original image was pixilated and each blue or white pixel was delivered as a different ICMS pattern to the Brainet during a series of trials (Stimulus 1 - white; Stimulus 2 - blue). The left panel shows the original input image and the right panel shows the output of the Brainet.

To improve the overall performance of this 4-rat Brainet, we implemented an adaptive decoding algorithm that analyzed the activity of each neuron in each specific bin separately, and then readjusted the neuronal weights following each trial (see Methods for details). Figure 4A depicts this Brainet architecture. Notice the different weights for each of the individual neurons (represented by different shades of grey), reflecting the individual accuracy in decoding the ICMS pattern. Figure 4B illustrates a session in which all four rats contributed to the overall decoding of the ICMS stimuli (the red color indicates periods of maximum decoding). Using this approach, we increased both the overall Brainet performance (74.18 ± 2.2% correct trials; n = 7 rats in 12 sessions) and the number of trials performed (64.17 ± 6.2 trials) in each session. The neuronal ensembles of this Brainet included an average of 50 ± 43 neurons (mean ± standard error). Figure 4C depicts the improved performance of the Brainet compared to that of the No-ICMS sessions (54.34 ± 2.2% correct trials, n = 11 sessions) and the performance of individual rats (61.28 ± 1.1% correct trials, F = 26.34; df = 2, 56; P < 0.0001; Bonferroni post hoc comparisons; Brainet vs No-ICMS: P < 0.0001; Brainet vs Individual rats P < 0.0001).

When rats were anesthetized (2 sessions in five rats) or trial duration was reduced to 10 s (i.e. almost only comprising the ICMS and the test period – 2 sessions in four rats), the Brainet’s performance dropped sharply (anesthetized: 60.61 ± 2.8% correct; short time trials: 62.57 ± 3.14%). Once again, this control experiment indicated that the Brainet operation was not solely dependent on an automatic response to the delivery of an ICMS.

Next, we investigated the dependence of the Brainet’s performance on the number of S1 neurons recorded simultaneously. Figure 4D depicts a neuron dropping curve illustrating this effect. According to this analysis, Brainets formed by larger cortical neuronal ensembles performed better than those containing just a few neurons^9^.

The difference between the Brainet classification of the two stimuli during regular sessions and during those in which no-ICMS was delivered is shown in Fig. 4E. During the regular sessions stimulus classification remained mostly in the quadrants corresponding to the stimuli delivered (lower left and upper right quadrants), while during the No-ICMS sessions the 4-rat Brainet trial classification was evenly distributed across all quadrants.

As different rats were introduced to the Brainet, we also compared how neuronal ensemble encoding in each animal changed during initial and late sessions (the first three versus the remaining days). Overall, there was a significant increase in ICMS encoding (initial: 59.67 ± 1.4%, late: 65.08 ± 1.2%, Mann-Whitney U = 281.0, P = 0.0344) and, to a smaller extent, in the correlation coefficients between neural activity of the different animals (initial: 0.1831 ± 0.007, late: 0.2028 ± 0.005, Mann-Whitney U = 275.0, P = 0.0153) suggesting that improvements in Brainet performances were accompanied by cortical plasticity in the S1 of each animal.

To demonstrate a potential application for this stimulus discrimination task, we tested whether our Brainet could read out a pixilated image (N = 4 rats in n = 4 sessions) using the same principles demonstrated in the previous two experiments. Blue and white pixels were converted into binary codes (white - Stimulus 1 or blue - Stimulus 2) and then delivered to the Brainet over a series of trials. The right panel of Fig. 4F shows that a 4-rat Brainet was able to capture the original image with good accuracy (overall 87% correct trials) across a period of four sessions.

### Brainet for storage and retrieval of tactile memories

**Figure 5:**
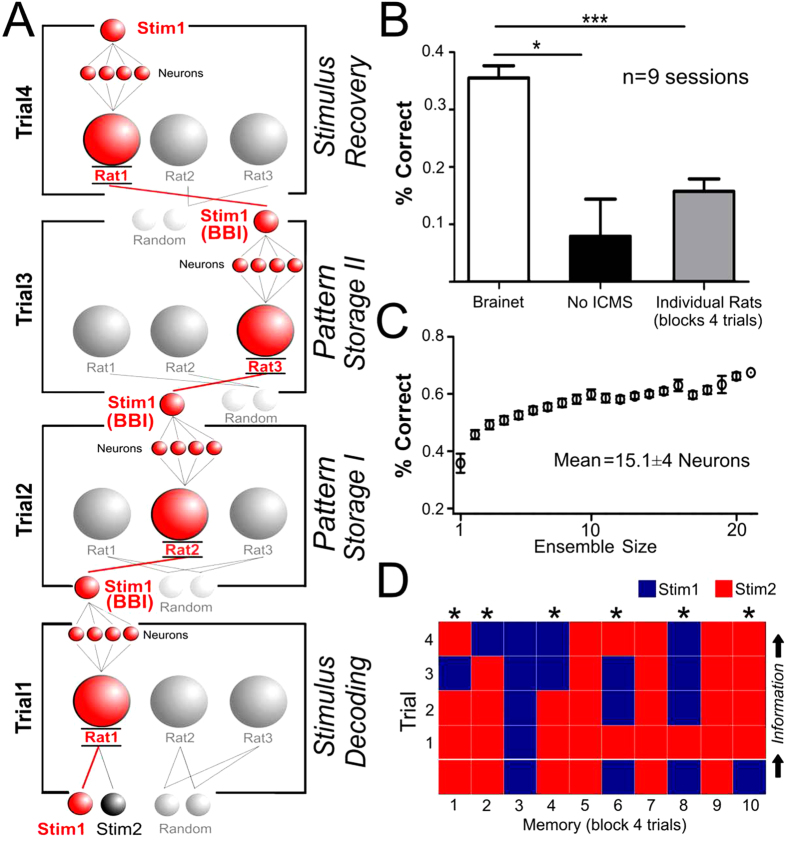
A Brainet for storage and retrieval of tactile memories. **A**) Tactile memories encoded as two different ICMS stimuli were stored in the Brainet by keeping information flowing between different nodes (i.e. rats). Tactile information sent to the first rat in Trial 1 (‘Stimulus Decoding’), was successively decoded and transferred between Rats 2 and 3, and again transferred to Rat 1, across a period of four trials (memory trace in red). The use of the brain-to-brain interface between the nodes of the network allowed accurate transfer of information. **B**) The overall performance of the Brainet was significantly better than the performance in the No-ICMS sessions and better than individual rats performing 4 consecutive correct trials. In this panel, * indicates *P* < 0.05 and *** indicates *P* < 0.001. **C**) Neuron dropping curve of Brainet for storage and retrieval of memories. D) Example of session with multiple memories (each column) processed in blocks of four trials (each row). Information flows from the bottom (Stimulus delivered) towards the top (Trials 1–4). Blue and red indicate Stimulus 1 or 2 respectively. Correct tactile memory traces are columns which have a full sequence of trials with the same color (see blocks: 3, 5, 7 and 9). In this panel, * indicates an incorrect trial.

To test whether a 3-rat Brainet could store and retrieve a tactile memory, we sent an ICMS stimulus to the S1 of one rat and then successively transferred the information decoded from that rat’s brain to other animals, via a BtBI, over a block of four trials. To retrieve the tactile memory, the information traveling across different rat brains was delivered, at the end of the chain, back to the S1 cortex of the first rat for decoding (Fig. 5A). Opaque panels were placed between the animals, and cortical neural activity was analyzed for each rat separately. The architecture of inputs and outputs of the 3-rat Brainet’s is shown in Fig. 5A, starting from the bottom shelf and progressing to the top one. The experiment started by delivering one of two different ICMS stimuli to the S1 of the input rat (from now on referred to as Rat 1) during the first trial (Trial 1). Neuronal ensemble activity sampled from Rat 1 was then used to decode the identity of the stimulus (either Stimulus 1 or 2). Once the stimulus identity was determined, a new trial started and a BtBI was employed to deliver a correspondent ICMS pattern to Rat 2, defining Trial 2 of the task. In this arrangement, the BtB link between Rat 1 and Rat 2 served to store the pattern (Pattern Storage I). Next, neuronal ensemble activity was recorded from the S1 of Rat 2. In the third trial, it was Rat 3’s turn to receive the tactile message (Pattern Storage II) decoded from the neural ensemble activity of Rat 2, via an ICMS mediated BtB link. During the fourth and final trial, Rat 1 received the message decoded from the neural activity of Rat 3.

Using this Brainet architecture, the memory of a tactile stimulus could only be recovered if the individual BtB communication links worked correctly in all four consecutive trials. The chance level for this operation was 6.25%. Under these conditions, this Brainet was able to retrieve a total of 35.37 ± 2.2% (9 sessions in 9 rats) of the tactile stimuli presented to it (Kruskall Wallis statistic = 14.89; P = 0.0006, Fig. 5B), contrasting with 7.91 ± 6.5% in No-ICMS sessions (n = 5 sessions; Dunn’s test: P < 0.001). For comparison purposes, individual rats performed the same four-trial task correctly in only 15.63 ± 2.1% of the trials. This outcome was significantly lower than a 3-rat Brainet (Dunn’s test: P < 0.001). As in the previous experiments, larger neuronal ensembles yielded better encoding (Fig. 5C).

As an additional control, rats that were not processing memory related information in a specific trial (e.g. Rats 2 and 3 during the Stimulus Decoding Stage in Rat 1) received Stimulus 1 or Stimulus 2, randomly chosen. Thus, in every single trial all rats received some form of ICMS, but only the information gathered from a specific rat was used for the overall tactile trace.

The colored matrix in Fig. 5D illustrates a session in which a tactile trace developed along the 3-rat Brainet. A successful example of information transfer and recovery is shown in the third block of trials (blue column on the left). The figure shows that the original stimulus (Stimulus 1 – bottom blue square) was delivered to the S1 of Rat 1 in the first trial. This stimulus was successfully decoded from Rat 1’s neural activity, as shown by the presence of the blue square immediately above it (Trial 1 – Stimulus Decoding). In Trial 2 (Pattern Storage I), Stimulus 2 was delivered, via ICMS to the S1 of Rat 2, and again successfully decoded (as shown by the blue square in the center). Then, in Trial 3 (Pattern Storage II), the ICMS pattern delivered to Rat 3 corresponded to Stimulus 1, and the decoding of S1 neural activity obtained from this animal still corresponded to Stimulus 1, as shown by the blue square. Lastly, in Trial 4 (Stimulus Recovery), Rat 1 received an ICMS pattern corresponding to Stimulus 1 and its S1 neural activity still encoded Stimulus 1 (blue square). Thus, in this specific block of trials, the original tactile stimulus was fully recovered since all rats were able to accurately encode and decode the ICMS pattern received. Similarly, columns 5, 7, and 9 also show blocks of trials where the original tactile stimulus (in these cases Stimulus 2, red square) was accurately encoded and decoded by the Brainet. Alternatively, columns with an asterisk on top (e.g. 1 and 8) indicate incorrect blocks of trials. In these incorrect blocks, the stimulus delivered was not accurately encoded in the brain of at least one rat belonging to the Brainet (e.g. rat 3 in block 1).

### Brainet for sequential and parallel processing

**Figure 6:**
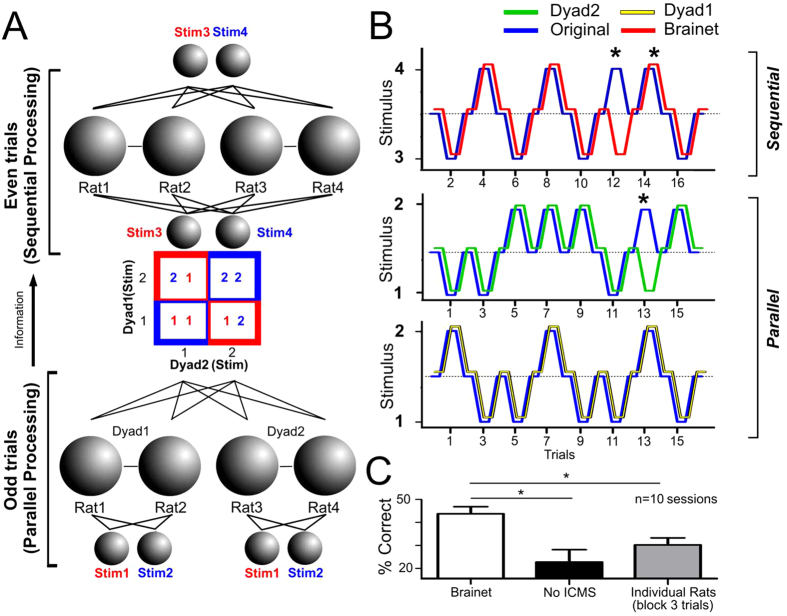
A Brainet for parallel and sequential processing. **A**) Architecture of a network for Parallel and Sequential processing. Information flows from the bottom to the top during the course of two trials. In first trial, odd trial for parallel processing, Dyad 1 (Rat 1-Rat 2) received one of two ICMS patterns, and Dyad 2 (Rat 3-Rat 4) received independently one of two ICMS patterns. During Trial 2, even trial for sequential processing, the whole Brainet received again one of two ICMS patterns. However, the pattern delivered in the even trial was dependent on the results of the first trial and was calculated according to the colored matrix presented. As depicted by the different encasing of the matrix (blue or red), if both dyads encoded the same stimulus in the odd trial (Stimulus 1-Stimulus1 or Stimulus 2-Stimulus 2), then the stimulus delivered in the even trial corresponded to Stimulus 3. Otherwise, if each dyad encoded a different stimulus in the odd trial (Stimulus1-Stimulus 2 or Stimulus 2-Stimulus 1), then the stimulus delivered in even trial was Stimulus 4. Each correct block of information required three accurate estimates of the stimulus delivered (i.e. encoding by both dyads in the even trial, as well as the whole Brainet in the odd trial). **B**) Example of session with sequential and parallel processing. The bottom and center panel show the dyads processing the stimuli during the odd trials (parallel processing), while the top panel shows the performance of the whole Brainet during the even trials. In this panel, * indicates an incorrect classification. **C**) The performance of the Brainet was significantly better than the performance during the No-ICMS sessions and above the performance of individual rats performing blocks of 3 correct trials. In this panel, * indicates *P* < 0.05.

Lastly, we combined all the processing abilities demonstrated in the previous experiments (discrete tactile stimulus classification, BtB interface, and tactile memory storage) to investigate whether Brainets would be able to use sequential and parallel processing to perform a tactile discrimination task (N = 5 rats in N = 10 sessions). For this we used blocks of two trials where tactile stimuli were processed according to Boolean logic^10^ (Fig.6A–B). This means that in each trial there was a binary decision tree (i.e. two options encoded as Stimulus 1 or 2). In the first trial, two different tactile inputs were independently sent to two dyads of rats (Dyad 1: Rat 1-Rat 2; Dyad 2: Rat 3-Rat 4; bottom of Fig. 6A). In the next trial, the tactile stimuli decoded by the two dyads were combined and transmitted, as a new tactile input, to a 4-rat Brainet. Upon receiving this new stimulus, the Brainet was in charge of encoding a final solution (i.e. identifying Stimulus 3 or 4, see Supplementary Figure 2).

As shown at the bottom of Fig. 6A, odd trials were used for parallel processing, i.e. each of two rat dyads independently received ICMS patterns, while neural activity was analyzed and the original tactile stimulus decoded (i.e. Stimulus 1 or 2). Then, during even trials (Fig. 6A, top), ICMS was used to encode a second layer of patterns, defined as Stimulus 3 and Stimulus 4. Note that ICMS Stimuli 3 and 4 were physically identical to Stimuli 2 and 1 respectively; however, because the stimuli delivered in the even trials were contingent on the results of the odd trials, we employed a different nomenclature to identify them. The decision tree (i.e. truth table) used to calculate the stimuli for the even trials is shown in the colored matrix at the center of Fig. 6A. The matrix shows that, if both dyads encoded the same tactile stimulus in the odd trial (Stimulus 1-Stimulus 1, or Stimulus 2-Stimulus 2; combinations with blue encasing), the ICMS delivered to the entire Brainet in the even trial corresponded to Stimulus 4. Otherwise, if the tactile stimulus decoded from each rat dyad in the odd trial was different (Stimulus 1-Stimulus 2, or Stimulus 2-Stimulus 1; combinations with red encasing), the ICMS delivered to the entire Brainet in the even trial corresponded to Stimulus 3. As such, the ICMS pattern delivered in even trials was the same for the whole Brainet (i.e. all four rats).

At the end of each even trial, the stimulus decoded from the combined neuronal activity of the four brain ensemble (top of Fig. 6A) defined the final output of the Brainet. Chance level was set at 12.5%. Overall, this Brainet performance was much higher than chance level or No-ICMS sessions (Brainet: 45.22 ± 3.4%, n = 10 sessions) significantly above No-ICMS sessions (n = 5 sessions) (No-ICMS: 22.79 ± 5.4%; Kruskal-Wallis statistic = 7.565, P = 0.0228; Dunn’s test: P < 0.05 Fig. 6C). Additionally, the Brainet also outperformed each individual rat (groups of three consecutive trials: 30.25 ± 3.0%; Dunn’s test: P < 0.05).

**Figure 7:**
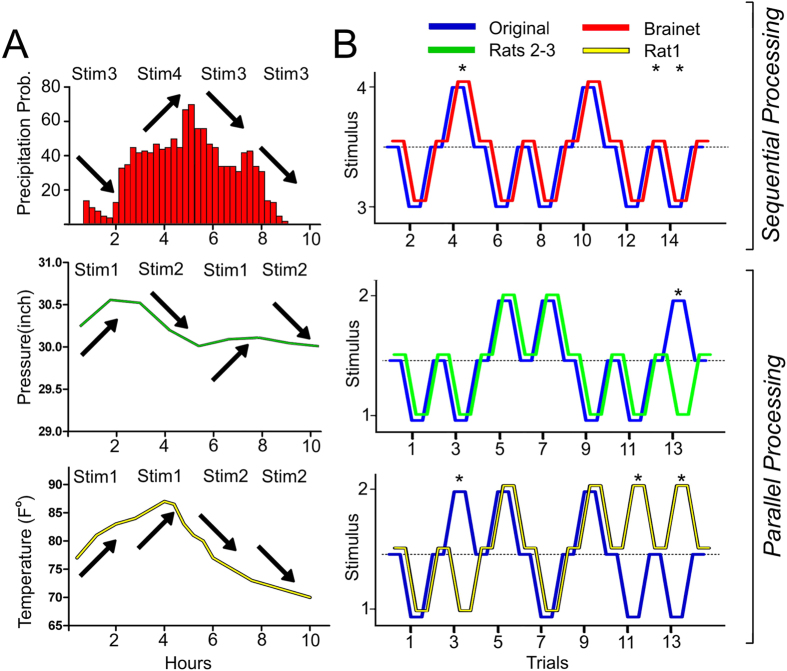
Parallel and sequential processing for weather forecast **A**) Each panel represents examples of the original data, reflecting changes in temperature (lower panel), barometric pressure (center panel), and probability of precipitation (upper panel). The arrows represent general changes in each variable, indicating an increase or a decrease. On the top of each panel is represented the ICMS pattern that resulted from each arrow presented. **B**) Lower and center panels show trials where different rats of the Brainet (Rat 1 lower panel, and Rats 2-3 center panel) processed the original data in parallel. Specifically, Rat 1 processed temperature changes and Rats 2-3 processed barometric pressure changes. The upper panel shows the Brainet processing changes in the probability of precipitation (Rats 1–3) during the even trials. * indicates trials where processing was incorrect.

As our last experiment, we tested whether a 3-rat Brainet could be used to classify meteorological data (see Methods for details). Again, the decision tree included two independent variables in the odd trials and a dependent variable in the even trials (see Supplementary Figure 3). Figure 7A illustrates how Boolean logic was applied to convert data from an original weather forecast model . In the bottom panel, the yellow line depicts continuous changes in temperature occurring during a period of 10 hours. Periods where the temperature increased were transferred to the Brainet as Stimulus 1 (see arrows in periods between 0 and 4 hours), whereas periods where the temperature decreased were transferred as Stimulus 2 (see arrows in periods between 6 and 10 hours). The middle panel of Fig. 7A illustrates changes in barometric pressure (green line). Again, periods where the barometric pressure increased were translated as Stimulus 1 (e.g. between 1-2 hours), while periods where the barometric pressure decreased were translated as Stimulus 2 (e.g. 3–5 hours).

Both Stimulus 1 and 2 were delivered to a Brainet during odd trials; changes in temperature were delivered to Rat 1 alone, while changes in barometric pressure were delivered to Rats 2 and 3. As in the previous experiment, Stimuli 3 and 4 were physically similar to Stimuli 1 and 2. In even trials, increases and decreases in the probability of precipitation (top panel Fig. 7A) were calculated as follows: an increase in temperature (Stimulus 1; Rat 1) combined with a decrease in barometric pressure (Stimulus 2; Rats 2 and 3) was transferred to even trials as an increase in the probability of precipitation (i.e. a Stimulus 4), whereas any other combination was transferred as Stimulus 3, and associated with a decrease in precipitation probability. This specific combination of inputs was used because it reflects a common set of conditions associated with early evening spring thunderstorms in North Carolina.

Overall, our 3-rat Brainet predicted changes in the probability of precipitation with 41.02 ± 5.1% accuracy which was much higher than chance (No-ICMS: 16.67 ± 8.82%; n = 3 sessions; t = 2.388, df = 4; P = 0.0377) (also see Fig. 7B).

## Discussion

In this study we described different Brainet architectures capable of extracting information from multiple (3-4) rat brains. Our Brainets employed ICMS based BtBs combined with neuronal ensemble recordings to simultaneously deliver and retrieve information to and from multiple brains. Multiple BtBIs were used to construct some of our Brainet designs. Our experiments demonstrated that several Brainet architectures can be employed to solve basic computational problems. Moreover, in all cases analyzed the Brainet performance was equal or superior to that of an individual brain. These results provide a proof of concept for the possibility of creating computational engines composed of multiple interconnected animal brains.

Previously, Brainets have incorporated only up to two subjects exchanging motor or sensory information^2^, or up to three monkeys that collectively controlled the 3D movements of a virtual arm^8^. These studies provided two major building blocks for Brainet design: (1) information transfer between individual brains, and (2) collaborative performance among multiple animal brains. Here, we took advantage of these building blocks to demonstrate more advanced Brainet processing by solving multiple computational problems, which included discrete classification, image processing, storage and retrieval of memories, and a simplified form of weather forecasting^1^,^2^,^8^. All these computations were dependent on the collective work of cortical neuronal ensembles recorded simultaneously from multiple animal brains working towards a common goal.

One could argue that the Brainet operations demonstrated here could result from local responses of S1 neurons to ICMS. Several lines of evidence suggest that this was not the case. First, we have demonstrated that animals needed several sessions of training before they learned to synchronize their S1 activity with other rats. Second, the decoding for individual neurons in untrained rats was close to chance levels. Third, attempts to make the Brainet work in anesthetized animals resulted in poor performance. Fourth, network synchronization and individual neuron decoding failed when animals did not attend to the task requirements and engaged in grooming instead. Fifth, removing the reward contingency drastically reduced the Brainet performance. Sixth, after we reduced trial duration, the decoding from individual neurons dropped to levels close to chance.

Altogether, these findings indicate that optimal Brainet processing was only attainable in fully awake, actively engaged animals, with an expectation to be rewarded for correct performance. These features are of utmost importance since they allowed Brainets to retain the computational aptitudes of the awake brain^11^ and, in addition, to benefit from emergent properties resulting from the interactions between multiple individuals^2^. It is also noteworthy to state that the Brainets implemented here only allowed partial social interactions between subjects (through the Plexiglas panels). As such, it is not clear from our current study, to what extent social interactions played (or not) a pivotal role in the Brainet performance. Therefore, it will be interesting to repeat and expand these experiments by allowing full social contact between multiple animals engaged in a Brainet operation. In this context, Brainets may become a very useful tool to investigate the neurophysiological basis of animal social interactions and group behavior.

We have previously proposed that the accuracy of the BtBI could be improved by increasing the number of nodes in the network and the size of neuronal ensembles utilized to process and transfer information^2^. The novel Brainet architectures tested in the present study support these suggestions, as we have demonstrated an overall improvement in BtBI performances compared to our previous study (maximum of 72% correct in the previous study versus maximum of 87% correct here)^2^. Since neuron dropping curves did not reach a plateau, it is likely that the performance of our Brainet architectures can be significantly improved by the utilization of larger cortical neuronal samples. In addition, switching between sequential and parallel processing modes, as was done in the last experiment, allowed the same Brainet to process more than two bits of information. It is important to emphasize, however, that the computational tasks examined in this study were implemented through Boolean logic^10^,^12^. In future studies we propose to address a new range of computational problems by using simultaneous analog and digital processing. By doing so, we intend to identify computational problems that are more suitable for Brainets to solve. Our hypothesis is that, instead of typical computational problems addressed by digital machines, Brainets will be much more amenable to solving the kind of problems faced by animals in their natural environments.

The present study has also shown that the use of multiple interconnected brains improved Brainet performance by introducing redundancy in the overall processing of the inputs and allowing groups of animals to share the attentional load during the task, as previously reported for monkey Brainets^8^. Therefore, our findings extended the concept of BtBIs by showing that these interfaces can allow networks of brains to alternate between sequential and parallel processing^13^ and to store information.

In conclusion, we propose that animal Brainets have significant potential both as a new experimental tool to further investigate system neurophysiological mechanisms of social interactions and group behavior, as well as provide a test bed for building organic computing devices that can take advantage of a hybrid digital-analogue architecture.

## Methods

All animal procedures were performed in accordance with the National Research Council’s Guide for the Care and Use of Laboratory Animals and were approved by the Duke University Institutional Animal Care and Use Committee. Long Evans rats weighing between 250–350 g were used in all experiments.

### Tasks of synchronization and desynchronization

Groups of four rats, divided in two pairs (dyads), were placed in two behavioral chambers (one dyad in each chamber). Rats belonging to the same dyad (i.e. inside the same chamber) could see each other through a Plexiglas panel, but not the animals in the other dyad. Each trial in a session consisted of four different periods: baseline (from 0–9 seconds), ICMS (9–11 seconds), test (11–12 seconds), and reward (13–25 seconds). During the baseline period no action was required from rats. During the ICMS period a pattern of ICMS (20 pulses, at 22–26 Hz, 10–100 uA) was delivered to all rats simultaneously. During the Test period, neural activity from all neurons recorded in each rat was analyzed and compared to the neural activity of all other animals as a population. Spikes from individual channels were summed to generate a population vector representing the overall activity which generally constitutes a good indicator of whisking and/or licking activity^14^. The population vectors for each of the four rats were then normalized. Lastly, we calculated the Pearson correlation between the normalized population vector of each rat and the general population of rats (the average of the neural population vectors from three remaining rats). During Pre-Sessions neural activity was analyzed in each trial, but no ICMS or water reward was delivered. During Sessions, neural activity was analyzed after the delivery of an ICMS stimulus and if the threshold for a correct trial was reached (at least three rats with R> = 0.2) then a water reward was delivered. During the Post-Sessions, neural activity was recorded and a water reward was delivered if animals reached the threshold for a correct trial, however no ICMS stimuli were delivered.

Additionally, we also tested the effect of ICMS alone and in anesthetized animals (Ketamine/Xylazine 100 mg/kg). During the synchronization/desynchronization task two different ICMS patterns were delivered: Stimulus 1 consisted of the same pattern that was used for the synchronization task and the threshold for a correct trial remained the same. Stimulus 2 consisted of two short bursts of ICMS (2 × 4 pulses, 22–26 Hz separated by 250 ms interval) and the threshold for a correct response was less than three rats reaching an R value of 0.2 during the testing period.

### Adaptive decoding algorithm

During the experiments where the adaptive decoding algorithm was used (discrete classification, tactile memory storage, sequential and parallel processing), the ICMS patterns remained as previously. Neural activity was separately analyzed for each neuron in each rat and 25 ms distributions were built and filtered with a moving average of 250 ms. The overall structure of the sessions included an initial period of 16–30 trials where Stimuli 1 and 2 were delivered to rats in order to build the distributions for each stimulus. The overall firing rate for each bin in the test period was then analyzed and, according to the probability distributions, a vote for Stimulus 1 or for Stimulus 2 was calculated. Bins with similar spike distributions for both stimuli were not analyzed. A final vote for each cell was then calculated, using the votes from all the bins that presented differences in the firing rate for the two stimuli. Lastly, the final votes for each cell in the population were filtered with a sigmoid curve. This filtering allowed the best encoding cells in the ensembles to contribute significantly more than other cells to the overall decision made by the Brainet made in each trial. Additionally, the weight of the cell population could be automatically adjusted at different intervals (e.g. every 10 or 15 trials).

For the image processing experiment, groups of four rats were tested. An original image was pixilated and converted into multiple trials. Each trial corresponded to a white (Stimulus 1) or blue (Stimulus 2) pixel in the original image. In each trial one of two different ICMS stimuli was delivered to the Brainet. After the neural activity from the Brainet was decoded, a new image corresponding to the overall processing by the Brainet was recreated.

### Memory storage experiment

For this specific experiment only three rats were used in each session and ICMS frequency patterns varied between 20–100 Hz. The number of pulses remained the same as in the previous experiments. Each memory was processed across a period of four trials which represented four different stages of a memory being processed: Stimulus delivery (Trial 1), Pattern Storage I (Trial 2), Pattern Storage II (Trial 3), and lastly, Stimulus Recovery (Trial 4). Information was initially delivered to the S1 cortex of the first rat (Rat 1) in the first trial – Stimulus Delivery. In Trial 2, information decoded from the cortex of Rat 1 was delivered as an ICMS pattern to the second rat (Rat 2) - Pattern Storage I. In Trial 3, information decoded from the S1 of Rat 2 was delivered to Rat 3 - Pattern Storage II. In Trial 4, neural activity decoded from the cortex of Rat 3 was decoded and delivered to the cortex of Rat 1 as a pattern of ICMS. Lastly, if the stimulus encoding and decoding was correct across all four trials (chance level of 6.25%) a memory was considered to be recovered. The overall number of memories decoded, the percent of stimuli decoded and the accuracy of the brain-to-brain interface information transfer were measured. As a control measure the Plexiglas panels separating the dyads were made opaque for this experiment. Additionally, as the tactile pattern was delivered to each rat in the specific memory stage (delivery, storage or recovery), a random Stimulus 1 or 2 was delivered to the remaining rats. This random stimulation of the remaining individuals ensured that, in each trial, rats could not identify whether or not they were participating in the tactile trace.

### Sequential and parallel processing experiment

Each block of information processing consisted of two trials: the first trial corresponded to parallel processing and the second trial corresponded to sequential processing. Two dyads of rats were formed: Dyad 1 (Rat 1-Rat 2) and Dyad 2 (Rat 3-Rat 4). During the first trial each dyad processed one of two ICMS stimuli independently of the other dyad. After the delivery of the ICMS stimuli to each dyad, neural activity was decoded and the stimulus for Trial 2 was computed from the results. If both dyads encoded a similar stimulus (Stimulus 1 - Stimulus 1, or Stimulus 2 - Stimulus 2), then the ICMS stimulus in Trial 2 was Stimulus 3. Otherwise, if the dyads encoded different ICMS stimuli (Stimulus 1 - Stimulus 2, or Stimulus 2 - Stimulus 1), then the ICMS stimulus in Trial 2 would be Stimulus 4. Stimuli 1 and 3 and Stimuli 2 and 4 had the exact same physical characteristics (number of pulses). During the second trial the same stimulus was delivered simultaneously to all four rats, and the Brainet encoded an overall response. A block of information was considered to be correct only if both Trials 1 and 2 were correct in both the dyads and in the Brainet.

For the weather forecasting experiment groups of three animals were tested. Sessions were run as described above for sequential and parallel processing. However, Trial one (parallel processing) was processed only by one rat (temperature) and one dyad of rats (barometric pressure), while Trial two (sequential processing: probability of precipitation) was processed by the whole Brainet (three rats).

To establish a simple weather forecast model we used original data from Raleigh/Durham Airport (KRDU), at WWW.Wunderground.com. Estimates were collected on August 2, 2014. We used periods characterized by increases and decreases in temperature and barometric pressure as independent variables, and increases in the probability of precipitation as the dependent variable. A total of 13 periods were collected. These included a total of 26 independent inputs for even trials (13 variations in temperatures and 13 variations in barometric pressure), as well as 13 additional changes in the probability of precipitation, to be compared with the Brainet outputs (i.e. the actual forecast). Specifically, for this experiment, increases in temperature (Stimulus 1 for the first rat) with decreases in barometric pressure (Stimulus 2 in Rats 2-3), during the odd trials, were computed as an increase in the probability of precipitation (Stimulus 4 to the Brainet in the even trial). Otherwise, increases or decreases in temperature (Stimulus 1 or 2 in the odd trial) combined with an increase in barometric pressure (Stimulus 1 for Rats 2 and 3), were computed as a decrease in the probability of precipitation (Stimulus 3 for the Brainet) in the even trial. Stimuli 1 and 3, and Stimuli 2 and 4 had the exact same physical characteristics (number of pulses).

### Surgery for microelectrode array implantation

Fixed or movable microelectrode bundles or arrays of electrodes were implanted bilaterally in the S1 of rats. Craniotomies were made and arrays lowered at the following stereotaxic coordinates: [(AP) −3.5 mm, (ML), ±5.5 mm (DV) −1.5 mm].

### Electrophysiological recordings

A Multineuronal Acquisition Processor (64 channels, Plexon Inc, Dallas, TX) was used to record neuronal spikes, as previously described^15^. Briefly, differentiated neural signals were amplified (20000–32,000×) and digitized at 40 kHz. Up to four single neurons per recording channel were sorted online (Sort client 2002, Plexon inc, Dallas, TX).

### Intracortical electrical microstimulation

Intracortical electrical microstimulation cues were generated by an electrical microstimulator (Master 8 , AMPI, Jerusalem, Israel) controlled by custom Matlab script (Nattick, USA) receiving information from a Plexon system over the internet. Patterns of 8–20 (bipolar, biphasic, charge balanced; 200 μsec) pulses at 20–120 Hz were delivered to S1. Current intensity varied from 10–100 μA.

## Additional Information

**How to cite this article**: Pais-Vieira, M. *et al.* Building an organic computing device with multiple interconnected brains. *Sci. Rep.* **5**, 11869; doi: 10.1038/srep11869 (2015).

## Supplementary Material

Supplementary Information
