# Corrigendum: Building an organic computing device with multiple interconnected brains

**DOI:** 10.1038/srep14937

**Published:** 2015-10-15

**Authors:** Miguel Pais-Vieira, Gabriela Chiuffa, Mikhail Lebedev, Amol Yadav, Miguel A. L. Nicolelis

Scientific Reports 5: Article number: 1186910.1038/srep11869; published online: 07092015; updated: 10152015

In this Article, an additional affiliation for Gabriela Chiuffa was omitted. The correct affiliation is listed below:

Center for Mathematics, Computation and Cognition, Federal University of ABC (UFABC), Sao Paulo, Brazil

In addition, the Acknowledgements section is incomplete.

“This work was supported by NIH R01DE011451, R01NS073125, RC1HD063390, National Institute of Mental Health award DP1MH099903, and by Fundacao BIAL 199/12 to MALN.”

should read:

“This work was supported by the CAPES Foundation to G.C.; and by NIH R01DE011451, R01NS073125, RC1HD063390, National Institute of Mental Health award DP1MH099903, and by Fundacao BIAL 199/12 to MALN.”

