# Supplementary material for: Building an organic computing device with multiple interconnected brains
Source: Sci Rep. 2015 Jul 9;5:11869. doi: 10.1038/srep11869 (PMC4497302; doi:10.1038/srep11869)
Supplement: Supplementary Information [file srep11869-s1.pdf]

Supplementary Materials

Building an organic computing device with multiple interconnected brains

Miguel Pais-Vieira[1], Gabriela Chiuffa[1], Mikhail Lebedev[1,4], Amol Yadav[2], and Miguel A.L. Nicolelis[1-5]

Departments of [1]Neurobiology, [2]Biomedical Engineering, and [3]Psychology and Neuroscience and [4]Center for Neuroengineering, Duke University, Durham, North Carolina 27710, and [5]Edmond and Lily Safra International Institute for Neuroscience of Natal, Natal, Brazil

Supplementary Figure 1

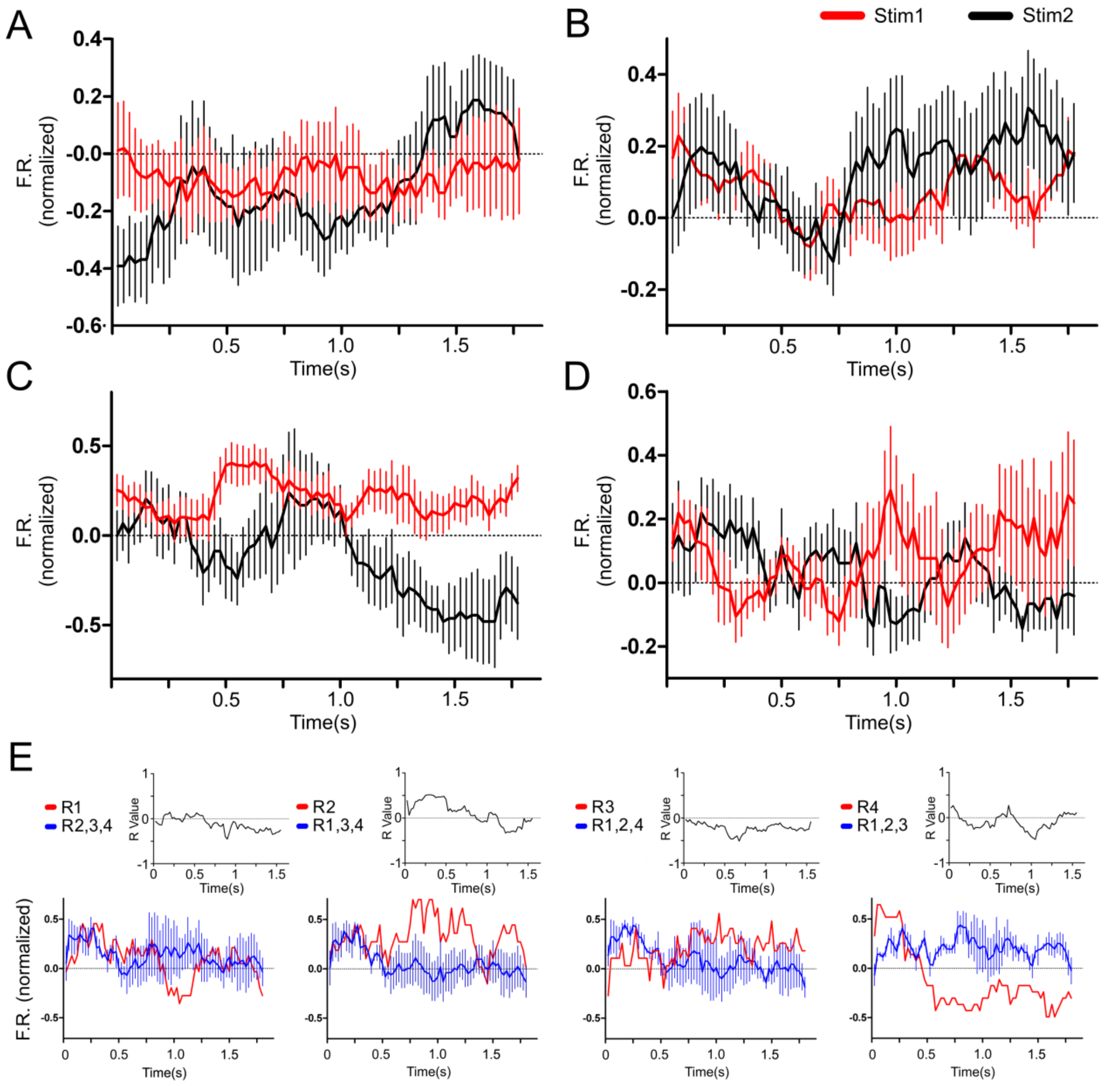

Examples of synchronized and desynchronized activity in the Brainet. A-D) Each panel depicts the mean of neural activity of each rat in the Brainet (Rats 1-4 in panels A-D respectively). Neural activity from trials where a Stimulus 1 was delivered is presented in red, while neural activity from trials where a Stimulus 2 was delivered is presented in black. While ICMS induced a fairly similar immediate response profile in many neurons, the overall ensemble response was also dependent on the animals' behavior. This absence

of an automatic response is clear in the four panels presented where the changes in the ensemble firing rate do not occur at the same time (compare black line in A and C in 0-250ms and 1000-1750). Similarly, in some animals the delivery of Stimulus 2 was associated with an overall increase in the neural ensemble firing rate (panel B) while in others it was associated with a local decrease (A and C). Values indicate mean±sem. E) Example of a desynchronized trial (Stimulus 2 delivered). The lower panels show, in red, the neural ensemble activity of each rat (red) and the average of neural ensemble activity for the remaining of the Brainet (blue). The upper panels depict the R value for the correlation coefficient between each rat and the remaining of the Brainet. Neural ensemble activity for two rats was correlated to the remainder of the Brainet (Rats 2 and 4) in this trial, but not in the other two (Rats 1 and 3). As the criterion for synchronization required at least three rats to be synchronized, this was a successful trial.

Supplementary Figure 2

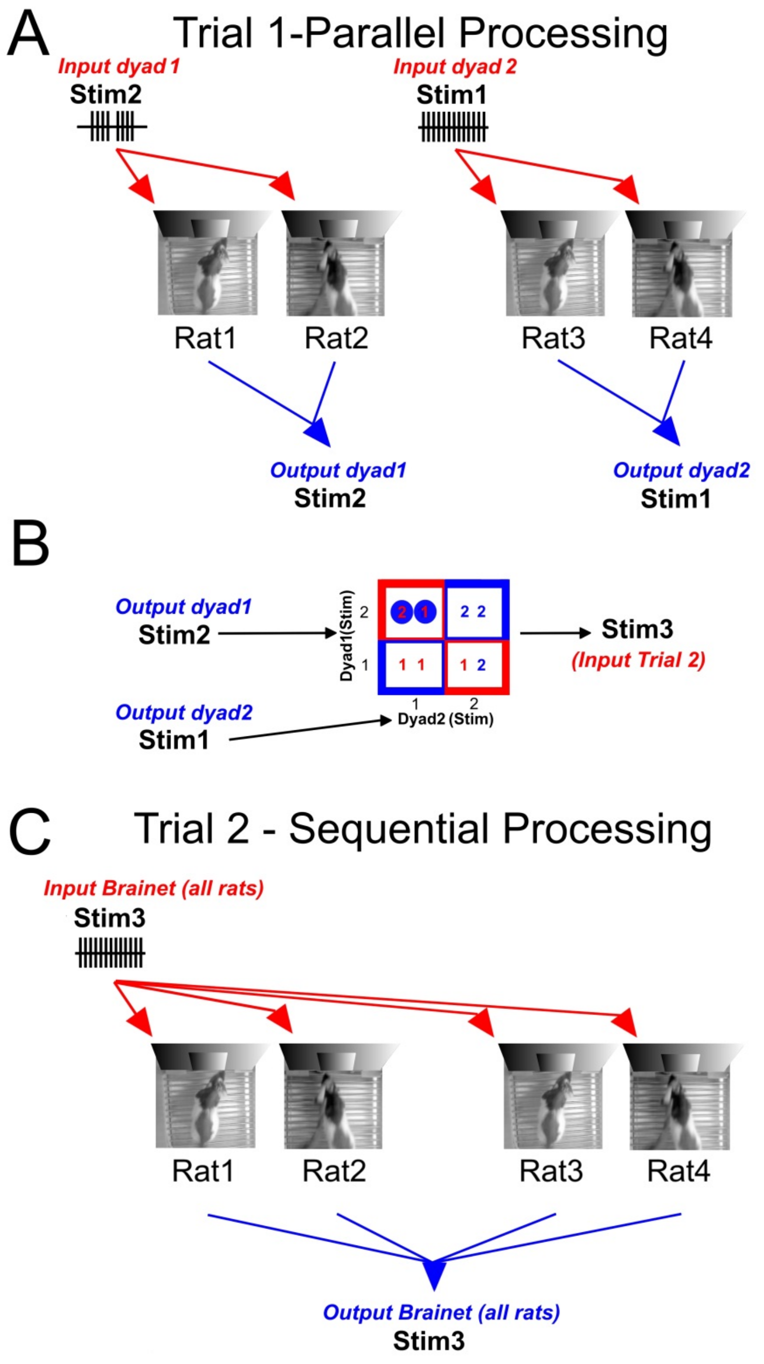

Example of brainet architecture for parallel and sequential processing. Information input is depicted by red arrows and information output is depicted with blue arrows. A) During odd trials, here represented as Trial 1, two independent inputs were sent to each dyad of rats. Here, Dyad 1 (Rats 1-2) received a Stimulus 2, while Dyad 2 (Rats 3-4) received Stimulus 1. Neural activity was recorded and analyzed in real time. Then, the identity of the stimulus delivered to the cortex was decoded and an output for each dyad was calculated. In this panel, neural activity from Dyad 1 encoded Stimulus 2 while the neural activity recorded from Dyad 2 encoded Stimulus 1. B) The outputs decoded from each dyad's neural activity were then processed according to the truth table presented. As shown by the different colors encasing the truth table, each combination of outputs from Trial 1 resulted in a particular stimulus being delivered in Trial 2. If similar stimuli were decoded during Trial 1, then they were transferred as a Stimulus 4 (represented by the combinations with blue encasing) during Trial 2. Otherwise, different stimuli were transferred as Stimulus 3 (represented by the combinations with red encasing). It is important to note that Stimulus 3 and Stimulus 4 had the exact same characteristics as those of Stimulus 1 and Stimulus 2. In this example, Dyad 1 and Dyad 2 encoded different stimuli (Stimulus 2 and Stimulus 1 respectively) and according to the truth table the stimulus transferred was Stimulus 3. As indicated in red, this is the stimulus that was then delivered as the input to the Brainet during Trial 2. C) During even trials, here represented as Trial 2, the same stimulus was delivered to all the rats in the Brainet. In this example, Stimulus 3 was delivered to all rats. Neural activity was again recorded and analyzed in real time. Then, the identity of the stimulus delivered to the Brainet was

decoded and an output for the whole Brainet was calculated. In this panel, neural activity from the Brainet encoded Stimulus 3.

Supplementary Figure 3

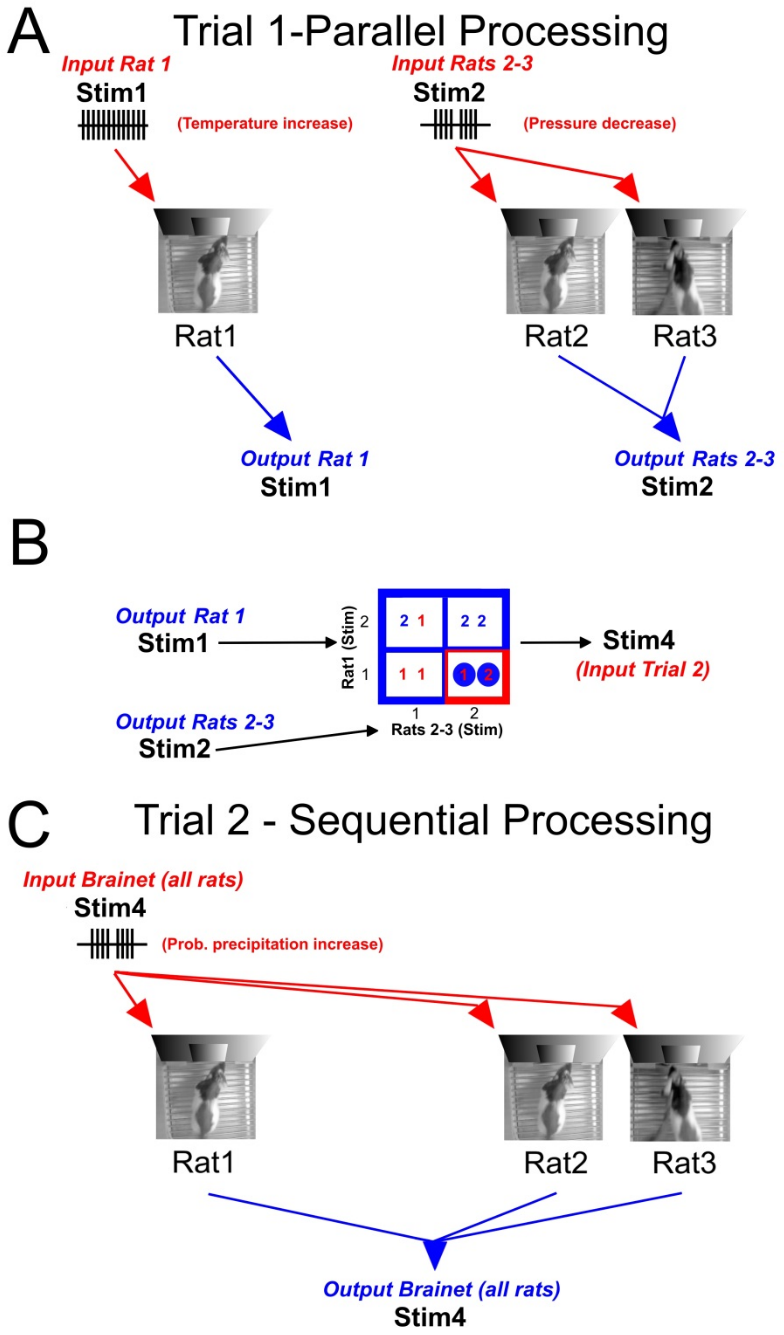

Example of brainet architecture for weather forecast. Information input is depicted by red arrows and information output is depicted with blue arrows. A) During odd trials, here represented as Trial 1, two independent inputs were sent to Rat 1 or to Rats 2-3. Here, Rat 1 received Stimulus 1 (which corresponded to a temperature increase in the original data), while Rats 2-3 received Stimulus 2 (which corresponded to a decrease in barometric pressure in the original data). Neural activity was recorded and analyzed in real time. Then, the identity of the stimulus delivered to the cortex was decoded and one output for Rat 1 and another for Rats 2-3 were calculated. In this panel, neural activity from Rat 1 encoded Stimulus 1 while the neural activity recorded from Rats 2-3 encoded Stimulus 2. B) The outputs decoded from the neural activity of Rat 1 and Rats 2-3 were then sent to the truth table. As shown by the different colors encasing the truth table, each combination of outputs from Trial 1 resulted in a particular stimulus being delivered in Trial 2. Note that this truth table is different from the one used in the previous experiment, since only a combination of Stimulus 1 in Rat 1 with Stimulus 2 in Rats 3-4 was transferred as Stimulus 4 (red encasing). Otherwise, all other combinations of stimuli were transferred as Stimulus 3 (blue encasing). ICMS Stimulus 3 and Stimulus 4 had the exact same characteristics that Stimulus 1 and Stimulus 2. In this example, Rat 1 and Rats 2-3 encoded Stimulus 1 and Stimulus 2 respectively, and according to the truth table the stimulus transferred was Stimulus 4. As indicated in red, this is the stimulus that was then delivered as the input for the Brainet during Trial 2. C) During even trials, here represented as Trial 2, the same stimulus was delivered to all the rats in the Brainet. In this example, Stimulus 4 was delivered to all rats. Neural activity was recorded and analyzed in real time. Then, the identity of the stimulus delivered to the Brainet was

decoded and an output for the whole Brainet was calculated. In this panel, neural activity from the Brainet encoded Stimulus 4, which indicated an increase in the probability of precipitation.
